# Supplementary material for: WordCommentsAnalyzer: A windows software tool for qualitative research
Source: F1000Res. 2018 Sep 4;7:536. Originally published 2018 May 3. [Version 2] doi: 10.12688/f1000research.14819.2 (PMC6137411; doi:10.12688/f1000research.14819.2)
Supplement: Supplementary file 3 [file f1000research-7-17614-s0002.tgz › b2a79496-4ada-47c4-9682-a99ce867836d.zip!/Eight Iranian Health Journals/Salmand_Iranian Journal of Ageing _8.docx]

© Salmand_Iranian Journal of Ageing . This work is licensed under CC BY-NC 4.0 (https://creativecommons.org/licenses/by-nc/4.0/).

Volume & Issue: دوره 11 - شماره 2

Title: رابطه مهارت‌های بینایی ساختاری و فعالیت‌های روزمره زندگی سالمندان مقیم جامعه

http://salmandj.uswr.ac.ir/article-1-1069-fa.html

اهداف: اختلالات شناختی، یکی از اختلالات شایع همراه با معلولیت شدید و پیش‌رونده در دوره سالمندی است. مطالعات نشان می‌دهد که حدود 5 درصد از افراد 65 سال و بالاتر به نقص شناختی واضح دچارند. از میان مهارت‌های شناختی، مهارت‌های ساختار بینایی یکی از پیش‌بینی‌کننده‌های مهم برای میزان دریافت کمک و حمایت در سالمندان محسوب می‌شود. کاهش بینایی به‌عنوان بخشی از شناخت، با اجرای فعالیت‌ها مرتبط است؛ بنابراین شناسایی این مشکلات در روند درمان و کسب استقلال در زندگی روزمره این افراد تأثیر بسزایی دارد. مواد و روش‌ها: در این مطالعه توصیفی-تحلیلی مقطعی 47 سالمند ساکن جامعه مراجعه‌کننده به کانون جهان‌دیدگان شهر شیراز در محدوده سنی 60 تا 80 سال به روش نمونه‌گیری دردسترس انتخاب شدند. اطلاعات از این طریق جمع‌آوری شد: مشاهده، مصاحبه، انجام خُرده‌آزمون مهارت‌های ساختار بینایی آزمون لوتکا برای ارزیابی مهارت‌های شناختی و شاخص کتز به‌منظور ارزیابی فعالیت‌های روزمره زندگی. همچنین برای تجزیه‌وتحلیل داده‌ها از نرم‌افزار SPSS و آزمون همبستگی پیرسون استفاده شد. یافته‌ها: شرکت‌کنندگان شامل 9 زن و 38 مرد با میانگین سنی 4/66±69/94 سال بودند. یافته‌های پژوهش نشان داد که سازمان‌دهی بینایی-حرکتی سالمندان ساکن جامعه با خُرده‌آزمون‌های غذاخوردن و لباس‌پوشیدن آزمون کتز ارتباط معنی‌داری دارد (0/05≥P). این در حالی است که بین خُرده‌آزمون بینایی-حرکتی آزمون لوتکا با خُرده‌آزمون‌های حمام‌کردن، جابه‌جایی در منزل، دست‌شویی‌رفتن و کنترل ادرار و مدفوع ارتباط معنی‌داری یافت نشد. همچنین بین نمره کلی آزمون کتز با خُرده‌آزمون بینایی حرکتی آزمون لوتکا نیز ارتباط معنی‌داری وجود نداشت (0/05). نتیجه‌گیری: براساس یافته‌های مطالعه حاضر، تنها بین اختلال در سازمان‌دهی بینایی-حرکتی و فعالیت لباس‌پوشیدن و غذاخوردن ارتباط معنی‌داری به‌دست آمد. به‌نظر می‌رسد شاخص کتز تنها به ارزیابی استقلال فردی در اجرای فعالیت‌های پایه روزمره زندگی می‌پردازد و نحوه اجرای این فعالیت‌ها را مدنظر قرار نمی‌دهد. ازآنجاکه فعالیت‌های پایه روزمره زندگی از جمله فعالیت‌هایی است که انسان پس از گذشت سال‌های اولیه زندگی به‌طورروزانه آنها را انجام می‌دهد، این ارتباط ضعیف بین شناخت و استقلال فردی را می‌توان با درنظرگرفتن اجرای این دسته از فعالیت ها به‌صورت عادت توجیه کرد. درحقیقت به‌دلیل آنکه فعالیت‌های پایه روزمره غالباً براساس عادت انجام می‌شود، نیاز کمتری به پردازش شناختی دارد؛ حال آنکه روند سالمندی بر نحوه انجام فعالیت‌ها تأثیر می‌گذارد و این تأثیر به‌گونه‌ای است که در بسیاری از مواقع با وجود آنکه فرد مرتکب خطاهای بسیاری می‌شود، درنهایت می‌تواند فعالیت را انجام دهد.

Title: رابطه سبک زندگی و حمایت اجتماعی با رضایت زناشویی سالمندان

http://salmandj.uswr.ac.ir/article-1-1018-fa.html

اهداف: با توجه به گسترش روزافزون جمعیت سالمندان در جوامع مختلف، به‌ویژه جامعه ایرانی، پژوهش حاضر با هدف بررسی رابطه سبک زندگی و حمایت اجتماعی با رضایت زناشویی در این گروه صورت گرفته است. مواد و روش‌ها: پژوهش حاضر از نوع پژوهش‌های توصیفی-همبستگی بود که به‌صورت مقطعی در سال 1393 انجام شد. جامعه آماری این پژوهش را تمامی سالمندان 60 سال و بالاتر تشکیل می‌داد که جزو جمعیت مراقبت‌شده بودند و در مراکز بهداشتی درمانی پرونده داشتند. نمونه آماری این مطالعه شامل 381 نفر از سالمندان بود که به روش نمونه‌گیری تصادفی خوشه‌ای چندمرحله‌ای از جامعه آماری پژوهش انتخاب شدند و به سؤالات مقیاس رضایت زناشویی انریچ، پرسش‌نامه سبک زندگی و حمایت اجتماعی پاسخ دادند. داده‌های گرد‌آوری‌شده با نسخه 21 نرم‌افزار SPSS و آزمون‌های ضریب همبستگی پیرسون و رگرسیون گام به‌گام مورد تجزیه‌وتحلیل قرار گرفت. یافته‌ها: نتایج بیانگر آن است که سبک زندگی و حمایت اجتماعی پیش‌بینی‌کننده رضایت زناشویی هستند. همچنین نتایج تحلیل رگرسیون گام‌به‌گام نشان داد که متغیر‌های پیش‌بین به‌ترتیب حمایت اجتماعی و سبک زندگی (0/64=R2) به‌شمار می‌روند. نتایج پژوهش حاضر بیانگر آن بود که بین متغیرهای سبک زندگی و حمایت اجتماعی با رضایت زناشویی رابطه معنی‌دار مثبت وجود دارد (0/05≥P)، اما بین مردان و زنان سالمند در این متغیرها تفاوت معنی‌داری وجود ندارد (0/05). نتیجه‌گیری: با توجه به تأثیر حمایت اجتماعی و سبک زندگی بر رضایت زناشویی سالمندان می‌توان گفت که حمایت اجتماعی و سبک زندگی عامل مهمی در پایداری ازدواج و رضایت زناشویی در این گروه اجتماعی است.

Title: اثربخشی درمان شناختی-رفتاری بر کیفیت خواب سالمندان مبتلا به اختلال بی‌خوابی

http://salmandj.uswr.ac.ir/article-1-989-fa.html

اهداف: با توجه به شیوع بالای اختلال بی‌خوابی در سالمندان، این پژوهش با هدف تأثیر درمان شناختی-رفتاری (CBT) بر کیفیت کلی خواب و خُرده‌مقیاس‌های کیفیت خواب سالمندان انجام شده است. مواد و روش‌ها: پژوهش حاضر از نوع آزمایشی بود و جامعه آماری پژوهش از 5000 سالمند عضو کانون جهان‌دیدگان شیراز و جامعه نمونه از 44 نفر با انتخاب تصادفی و احتساب شرایط ورود و خروج تشکیل می‌شدند. به‌دنبال ریزش 85/7 درصدی، 39 سالمند با میانگین سنی 68 سال که مبتلا به بی‌خوابی اولیه بودند، با شاخص کیفیت خواب پیتزبورگ (PSQI) در پیش‌آزمون، پس‌آزمون و پیگیری موردارزیابی قرار گرفتند. مداخله چهارهفته‌ای (دوبار در هفته) با روش درمان شناختی-رفتاری براساس دستورالعمل اسپای انجام شد. برای تجزیه‌وتحلیل داده‌ها از نسخه 21 نرم‌افزار آماری SPSS و روش‌های آماری تحلیل کوواریانس تک و چندمتغیره (آنکوا و مانکوا) استفاده شد. یافته‌ها: میانگین نمرات کیفیت کلی خواب قبل از مداخله در گروه‌های آزمایش و کنترل به‌ترتیب 12/95 و 12/7 بود که پس از مداخله در پس‌آزمون به 10/03 و 13/07 و در پیگیری سه ماه بعد به 9/51 و 13/36 تغییر یافت. تفاوت میانگین نمرات کیفیت کلی خواب پس از مداخله، از لحاظ آماری معنادار (0/001>P) بود. نتیجه‌گیری: براساس یافته‌های این پژوهش درمان شناختی-رفتاری، کیفیت کلی خواب سالمندان را ارتقا و نشانه‌های اختلال بی‌خوابی در آنها را کاهش می‌دهد. علاوه‌براین توصیه می‌شود درمانگران و متخصصان بهداشت روان در حیطه سالمندی از این روش استفاده کنند.

Title: بررسی تأثیر آرام‌سازی پیش‌رونده عضلانی بر عملکرد اجتماعی و ابعاد مختلف کیفیت زندگی سالمندان

http://salmandj.uswr.ac.ir/article-1-954-fa.html

اهداف: با پیشرفت علم افراد مدت طولانی‌تری زندگی می‌کنند و به‌ناچار باید با تغییرات پیچیده جسمی، روانی و اجتماعی سازگار شود که همراه با فرایند پیری ایجاد می‌شود. عوامل متعددی از جمله تحرک و ورزش بر فرایند پیری تأثیر دارد؛ به‌نحوی‌که ورزش منجر به ایجاد شادابی، نشاط و افزایش امیدبه‌زندگی می‌شود. این مطالعه با هدف بررسی میزان تأثیر آرام‌سازی پیش‌رونده عضلانی بر عملکرد اجتماعی و ابعاد مختلف کیفیت زندگی سالمندان شهرستان شهرکرد انجام گرفت. مواد و روش‌ها: پژوهش حاضر یک کارآزمایی بالینی بود که در آن برای جمع‌آوری داده‌ها از روش نمونه‌گیری تصادفی منظم استفاده شد. در این پژوهش، شصت نفر سالمند از شهرستان شهرکرد انتخاب شدند و نمونه‌ها به‌صورت تصادفی به دو گروه آزمون و شاهد تقسیم شدند. روش آرام‌سازی به این ترتیب بود که بیمار در هر مرحله، گروهی از عضلات بدن خود را منقبض و پس از پنج ثانیه رها می‌کرد و در پایان، تمام عضلات را شُل و پنج بار نفس عمیق می‌کشید. هر جلسه حدود 45 دقیقه به‌طول می‌انجامید. در گروه آزمون روش آرام‌سازی پیش‌رونده عضلانی به‌مدت سه ماه و سه روز در هفته انجام شد. به‌طورکلی مددجو پس از پوشیدن لباس راحتی به‌پشت می‌خوابید و با هدایت مربی آموزشی و گوش‌دادن به نوار صوتی، گروه‌های مختلف عضلانی را با تنفس‌های مؤثر و عمیق منقبض و آرام می‌کرد؛ به‌طوری‌که آرام‌شدن عضلانی را احساس و آن را بیان می‌کرد. ابزار گردآوری داده‌های این مطالعه پرسش‌نامه اطلاعات فردی و پرسش‌نامه کیفیت زندگی SF-36 بود.  یافته‌ها: نتایج مطالعه نشان داد آرام‌سازی پیش‌رونده عضلانی، منجر به افزایش عملکرد اجتماعی و ابعاد مختلف کیفیت زندگی در گروه مداخله می‌شود. بین میانگین نمره ابعاد عملکرد جسمی، محدودیت فعالیت به‌دنبال مشکل جسمی، انرژی و خستگی، عملکرد اجتماعی، درد بدنی، سلامت عمومی با کیفیت زندگی بین گروه مورد و شاهد تفاوت آماری معنی‌داری وجود دارد. همچنین میان عملکرد اجتماعی در دو گروه شاهد و مداخله قبل از مطالعه، تفاوت آماری معنی‌داری نبود؛ ولی بعد از مداخله عملکرد اجتماعی گروه مداخله تغییر یافته بود. نتیجه‌گیری: با توجه به نتایج این مطالعه توصیه می‌شود در زندگی روزانه برنامه‌ای برای انجام تمرینات ورزشی و آرام‌سازی پیش‌رونده عضلانی متناسب با وضعیت جسمانی سالمندان طراحی شود تا مدت‌زمان ناتوانی، روزهای بستری و هزینه‌های درمانی کاهش و عملکرد اجتماعی، کیفیت زندگی و وضعیت روحی-روانی سالمندان ارتقا پیدا کند. همچنین استقلال سالمند در انجام کارهای روزانه بهبود یابد و درنهایت، به فرایند پیری موفق و سالم آنان کمک کند.

Title: بررسی وضعیت سلامت سالمندان در روستاهای آشتیان: استان مرکزی، 1393

http://salmandj.uswr.ac.ir/article-1-945-fa.html

اهداف: با افزایش امیدبه‌زندگی و افزایش سن تراکم جمعیتی در برخی جوامع، جمعیت سالمندان جهان به‌طور روزافزونی زیاد می‌شود. بالارفتن سن،کاهش تدریجی فعالیت‌های فیزیکی و افزایش شیوع بیماری‌های مزمن در سالمندان می‌تواند به کاهش عملکرد فردی و اجتماعی آنها بیانجامد. از نکات مهم در ضرورت انجام این تحقیق می‌توان به این موارد اشاره کرد: تغییر الگوی اپیدمیولوژیک بیماری‌ها در سنین سالمندی، گرایش به‌سوی بیماری‌های مزمن و رویارویی با سالمندان برخوردار از سلامت نسبی ولی نیازمند مراقبت‌های بهداشتی‌درمانی. به‌منظور مدیریت بهینه و برنامه‌ریزی دقیق برای سلامت سالمندان روستایی، پژوهش در این زمینه ضروری است. هدف از انجام این مطالعه بررسی وضعیت سلامت و بیماری سالمندان روستاهای شهرستان آشتیان بود. مواد و روش‌‌‌‌‌ها: این پژوهش، مطالعه‌ای مقطعی از نوع توصیفی-تحلیلی بود که روی 200 نفر از سالمندان روستاهای آشتیان انجام شد. جمعیت هدف به روش خوشه‌ای انتخاب و برای تمام سالمندان پس از اخذ رضایت، فرم اطلاعاتی با پرسش و معاینه و پاسخ اقدامات پاراکلینیکی تکمیل شد. اطلاعات جمع‌آوری‌شده با استفاده از نرم‌افزار SPSS پردازش  و از آزمون کای اسکوئر استفاده و معنی‌داری 0/05>P  درنظرگرفته شد. یافته‌ها: در این مطالعه از 126 نفر زن و 74 نفر مرد، 156 نفر (78درصد) بی‌سواد، 52 نفر (26درصد) بیوه و دو نفر (1درصد) مجرد و 32 نفر (16درصد) تنها و 10 نفر (5درصد) سیگاری بودند. در این بررسی فراوانی برخی بیماری‌ها بدین‌شرح به‌دست آمد: استئوآرتریت 163 نفر (81/5درصد)، نداشتن تعادل 138 نفر (69درصد)، فشارخون بالا 120 نفر (60درصد)، چربی خون بالا 111 نفر (55/5درصد)، اختلالات ادراری 99 نفر (49/5درصد)، افسردگی 95 نفر (47/5درصد)، اختلالات شناختی 92 نفر (46درصد)، مشکلات شنوایی 88 نفر (44درصد)، مشکلات تغذیه‌ای 83 نفر (41/5درصد)، اختلالات خواب 76 نفر (38درصد)، مشکلات قلبی-عروقی 67 نفر (33/5درصد)، مبتلایان به دیابت 18 نفر (9درصد). درضمن افسردگی با بیوه‌بودن (0/027>P)، بی‌سوادی (0/001>P)، تنها زندگی‌کردن (0/025>P) و جنس مؤنث (0/001>P) ارتباط معنی‌دار نشان داد. نتیجه‌گیری: یافته‌ها نشان داده که بیماری‌ها و محدودیت‌های جسمانی و فیزیکی سالمندان ساکن روستاها از اهمیت فراوانی برخوردار است. در این پژوهش بالابودن درصد فراوانی بیماری‌های مزمن به‌ویژه بیماری‌های استئوآرتریت، نداشتن‌تعادل، افسردگی، فشارخون، مشکلات تغذیه‌ای، مشکلات بینایی و شنوایی و بیماری قلبی-عروقی در سالمندان ساکن روستا مشخص شد که در برنامه‌ریزی‌های بهداشتی باید مدنظر قرار گیرد. این مطالعه نشان داد زنان و سالمندان کم‌سواد و بیکار و سالمندانی که در تجرّد یا تنها در روستا زندگی می‌کنند با مشکلات جسمانی بیشتری مواجه هستند. ازاین‌رو توصیه می‌شود که در سیاست‌گذاری بهداشتی توجه ویژه‌ای به این گروه‌های خاص شود.

Title: بررسی روایی و پایایی پرسش‌نامه توانمندی سالمندان مبتلا به بیماری‌های قلبی - عروقی در دریافت حمایت اجتماعی

http://salmandj.uswr.ac.ir/article-1-939-fa.html

اهداف: افزایش مشکلات دوران سالمندی به‌همراه اختلالات قلبی-عروقی، توانمندی سالمندان را در مدیریت زندگی فردی برای بهره‌مندی از حمایت‌های اجتماعی تحت‌تأثیر قرار می‌دهد. بیماری‌های قلبی-عروقی بار اقتصادی و اجتماعی و روحی‌-روانی را به‌صورت هزینه‌های مستقیم بهداشتی و هزینه‌های غیرمستقیم دربردارد که از توانمندی و کیفیت زندگی افراد می‌کاهد. در این راستا طراحی ابزاری روا و پایا برای سنجش توانمندی سالمندان مبتلا به بیماری‌های قلبی-عروقی در دریافت حمایت‌های اجتماعی حائز اهمیت است. مواد و روش‌ها: در این مطالعه توصیفی‌، طراحی و روان‌سنجی پرسش‌نامه طی چهار مرحله مطابق روش والتز انجام شد: 1. تعریف مفهوم توانمندی سالمندان مبتلا به بیماری‌های قلبی-عروقی در دریافت حمایت اجتماعی با مروری بر کتب و مقالات؛ 2. طراحی گویه‌های پرسش‌نامه با استفاده از منابع موجود در ایران و دیگر کشور‌ها؛ 3. روان‌سنجی، اعتبار محتوا و اعتبار صوری پرسش‌نامه توسط 10 نفر از متخصصان دانشگاه؛ 4. بررسی همسانی درونی پرسش‌نامه با تعیین ضریب آلفای کرونباخ و پایایی ثبات با استفاده از آزمون مجدد و با فاصله زمانی یک هفته. یافته‌ها: با توجه به یافته‌های به‌دست‌آمده از مطالعه مقاله‌ها و کتاب‌ها و نظریه‌های توانمندسازی خودمدیریتی روانی‌پور و همکاران، توانمندی سالمندان مبتلا به بیماری‌های قلبی-عروقی در دریافت حمایت اجتماعی در محورهای خودآگاهی از تغییرات، عملکرد نقش، سازگاری، استقلال، رضایت پنداشت‌شده، حس کنترل و خودمدیریتی بود. نسخه ابتدایی پرسش‌نامه با 35 گویه طی مرحله اول و دوم مطالعه تهیه شد. در مرحله سوم و چهارم مطالعه طی مراحل روان‌سنجی و روایی و پایایی 4 گویه حذف شد. سپس نسخه نهایی پرسش‌نامه با 31 گویه و شاخص روایی محتوایی 0/94 و نسبت روایی محتوایی 0/96 تعیین شد. برای تعیین پایایی درونی پرسش‌نامه از ضریب آلفای کرونباخ استفاده شد که میزان آن 0/90 به‌دست آمد. همچنین برای تعیین پایایی بیرونی پرسش‌نامه از روش بازآزمایی استفاده شده که در دو مرحله به فاصله یک هفته تکمیل شد. نتایج نشان داد که از ضریب همبستگی مطلوب (0/83=R) برخوردار است. نتیجه‌گیری: نتایج مطالعه نشان داد پرسش‌نامه تدوین‌شده با 31 گویه، ویژگی‌های روان‌سنجی مطلوبی برای سنجش توانمندی سالمندان مبتلا به بیماری‌های قلبی-عروقی در دریافت حمایت‌های اجتماعی دارد. علاوه‌براین مراقبان امور سلامت می‌توانند این پرسش‌نامه را در سیستم‌های مراقبتی و بهداشتی کشور به‌کار گیرند. همچنین پرسش‌نامه‌ ذکرشده برای تعیین اثر مداخلات در زمینه توانمندی سالمندان در دریافت حمایت اجتماعی در تحقیقات کارآزمایی بالینی مناسب است. در پژوهش‌های بعدی پیشنهاد می‌شود انواع دیگر روایی مانند روایی سازه برای تعیین ابعاد توانمندی اندازه‌گیری شود.

Title: تأثیر یک برنامه تمرین قدرتی و تعادلی بر عملکرد تعادل و کیفیت زندگی مردان سالمند دارای ترس از افتادن

http://salmandj.uswr.ac.ir/article-1-566-fa.html

اهداف: افتادن‌ها در سالمندان عمدتاً به‌دلیل نقایص تعادل و ناپایداری قامتی رخ می‌دهد. افتادن‌ها و آسیب‌های ناشی از آنها به ترس از افتادن در سالمندی منجر می‌شود. این ترس رایج‌ترین ترس در سالمندان است و به اُفت خودتحمیل‌شده در فعالیت‌ها و کارکرد در سالمندی منجر می‌شود. افتادن‌ها و ترس از افتادن و پیامدهای منفی آنها می‌تواند اثراتی منفی بر کیفیت زندگی و فعالیت‌های زندگی روزانه و کارکردهای جسمانی داشته باشد. براین‌اساس هدف این پژوهش بررسی تأثیر برنامه مداخله تمرینی قدرتی و تعادلی روی عملکرد تعادل و کیفیت زندگی سالمندان دارای ترس از افتادن ساکن در جامعه بود. مواد و روش‌ها: روش این تحقیق، نیمه‌تجربی با طرح پیش‌آزمون و پس‌آزمون بود و جامعه آماری آن را سالمندان مرد شهر اراک تشکیل می‌دادند. از این جامعه 130 سالمند مرد در آزمون غربالگری شرکت کردند و از بین آنها 31 نفر واجد شرایط دارای ترس از افتادن انتخاب و به‌طورتصادفی به گروه آزمایش (51=n) با میانگین سنی 66/07 سال و گروه کنترل (61=n) با میانگین سنی 70/14 سال تقسیم شدند. آزمودنی‌های گروه آزمایش در برنامه مداخله تمرینی ده‌هفته‌ای، هر هفته سه جلسه و هر جلسه 90 دقیقه شرکت کردند. این مداخله تمرینی شامل تمرین کششی، تمرین قدرتی اندام‌های فوقانی و تحتانی، تمرین تعادل و سردکردن بود که در حضور یک پزشک اجرا شد. داده‌ها با استفاده از ابزارهای روا و پایای آزمون مختصر وضعیت روانی (MMSE)، کیفیت زندگی (SF-36)، اعتماد به تعادل ویژه فعالیت‌ها (ABC) و آزمون عملکردی تعادلی ایستادن یک‌پا جمع‌آوری شد. برای تجزیه‌و‌تحلیل داده‌ها از آزمون تی-استیودنت مستقل با سطح معنی‌داری 0/05 و نسخه 16 نرم‌افزار SPSS و نمره‌های پیش‌آزمون و پس‌آزمون از آزمون تی مقایسه‌ای استفاده شد.   یافته‌ها: بین میانگین نمره آزمون مختصر وضعیت روانی آزمودنی‌های گروه آزمایش و کنترل تفاوت معنی‌داری وجود نداشت (0/244=P). یافته‌ها نشان داد میانگین نمره آزمون عملکرد تعادل ایستادن یک‌پا با پای اتکا (0/039=P) و راهنما (0/001≥P) به‌طورمعنی‌داری بهبود یافت. به‌علاوه این برنامه مداخله تمرینی میانگین نمره زیرمقیاس‌های کیفیت زندگی از جمله کارکرد جسمانی، محدودیت نقش به‌دلیل سلامت جسمانی، درد بدنی، ادراک از سلامت عمومی، سرزندگی (انرژی/خستگی) و سلامت ذهنی عمومی را به‌طورمعنی‌داری بهبود داد (0/05≥P). در میانگین نمره زیرمقیاس‌های کارکرد اجتماعی و محدودیت نقش به خاطر مشکلات احساسی، پس از برنامه مداخله تمرینی تفاوت معنی‌داری مشاهده نشد (0/05<P). نتیجه‌گیری: براساس نتایج، 10 هفته برنامه مداخله تمرینی قدرتی و تعادلی، تعادل و کیفیت زندگی سالمندان دارای ترس از افتادن را بهبود بخشید. این نتایج نقش قدرت عضلانی و تعادل را در فعالیت‌های زندگی روزانه سالمندان دارای ترس از افتادن برجسته می‌کند. همچنین این یافته‌ها بر اهمیت استفاده از شیوه ترکیب مداخله تمرینی قدرتی و تعادلی برای بهبود وضعیت سلامتی و پیشگیری از افتادن در سالمندان تأکید می‌کند. بنابراین این برنامه می‌تواند در بهبود تعادل و کیفیت زندگی مرتبط با سلامت سالمندان مفید باشد و سالمندی موفقیت‌آمیز را ارتقا دهد.

Title: داروهای مناسب تجویزنشده در بیماران سالمند بستری در بیمارستا‌ن‌های منتخب دانشگاه علوم پزشکی ایران با استفاده از ابزار غربالگری داروهای مناسب تجویزنشده در افراد سالمند

http://salmandj.uswr.ac.ir/article-1-1098-fa.html

اهداف: افزایش جمعیت سالمندان تشویشی جهانی است و مراقبت از سلامت و کیفیت زندگی سالمندان بالای 65 سال، از مشکلات عمده امروز و آینده به‌شمار می‌آید. تجویز دارو جزئی اساسی از مراقبت بیماران سالمند و بهینه‌سازی درمان دارویی برای بیماران سالمند مسئله‌ساز است. این امر می‌تواند عوارض جانبی و هزینه‌ها را افزایش دهد. هدف از انجام این مطالعه بررسی وضعیت داروهای مناسب تجویزنشده در بیماران سالمند60 ساله و بالاتر با استفاده از ابزار غربالگری داروهای مناسب تجویزنشده در افراد سالمند (START) بود که در بیمارستان‌های آموزشی و غیرآموزشی دانشگاه علوم پزشکی ایران بستری شده بودند.  مواد و روش‌ها: این مطالعه مقطعی در چهار بیمارستان آموزشی و غیرآموزشی دانشگاه علوم پزشکی ایران به‌منظور بررسی میزان داروهای مناسب تجویزنشده براساس معیار START انجام شد. داده‌های پژوهش حاضر از پرونده 400 بیمار 60 ساله و بالاتر از بخش‌های بستری مشترک (سی.سی.یو، ایی.ان.تی و آی.سی.یو عمومی، ارتوپدی ، جراحی عمومی و داخلی) در طی شش‌ماه (از تیرماه تا آذرماه) سال 1392 جمع‌آوری شد که به‌صورت تصادفی انتخاب شده بودند. تجزیه‌وتحلیل داده‌ها با استفاده از نسخه 18 نرم‌افزار SPSS و نسخه 2013 نرم‌افزار اکسل انجام شد. همچنین برای تعیین ارتباط بین متغیرهای بررسی‌شده آزمون کای اسکوئر، من-ویتنی، ضریب همبستگی اسپیرمن و کلموگروف-اسمیرنف به‌کاررفت.  یافته‌ها: میانگین سن سالمندان مطالعه‌شده 73/4 سال بود. از بین سالمندان بررسی‌شده، بیماری قلبی-عروقی بیشترین درصد (21/6درصد) و بیماری دیابت کمترین درصد فراوانی (3/0) را در بین بیماری‌های فعلی سالمندان کسب کرده بودند. تعداد کل داروهای تجویزشده 4744 بود که برای 39/6درصد از سالمندان حداقل یک داروی مناسب تجویزنشده بود. بیشترین طبقات دارویی مناسب تجویزنشده براساس معیار START به‌ترتیب در بیمارستان‌های غیرآموزشی 11/7درصد بیس‌فسفوناتس و 8/3درصد آنتی‌دیابتیک و در بیمارستان‌های آموزشی 13/3درصد بیس‌فسفوناتس و 11/4درصد آنتی‌دیابتیک بوده است. میانگین داروی مناسب تجویزنشده رابطه معنی‌داری با طول مدت اقامت بیماران در بیمارستان‌های آموزشی داشته است. تعداد داروهای مناسب تجویزنشده با افزایش تعداد داروهای تجویزشده به‌صورت معنی‌داری افزایش یافته است. نتیجه‌گیری: با توجه به اینکه مسائل مربوط به میزان داروهای مناسب تجویزنشده در میان سالمندان بررسی‌شده می‌تواند جدی باشد؛ بنابراین تجویز داروهای مناسب برای سالمندان از اهمیت زیادی برخودار است. نظارت بر تجویز، نحوه تأمین و مصرف منطقی دارو بسیار حیاتی و اثرگذار است. ازآنجاکه بخشی از بودجه سلامت به دارو تعلق دارد، بیمارستان‌ها می‌تواند با بهره‌گیری از پزشکان شاغل به‌همراه دیدگاه‌های تخصصی مشاوران دارویی و داروسازان بالینی در کاهش این مشکل کارساز باشد.

Title: بررسی روانی واجی و روانی معنایی در سالمندان سالم فارسی‌زبان

http://salmandj.uswr.ac.ir/article-1-1067-fa.html

اهداف: اختلالات شناختی و زبانی از جمله مشکلات واژه‌یابی در سالمندان شایع است. روانی کلامی یکی از اولین کارکردهای شناختی است که در جریان فرایند سالمندی زوال می‌یابد. با توجه به نبودِ هنجارهای روانی کلامی در سالمندان طبیعی فارسی‌زبان، هدف مطالعه حاضر بررسی روانی کلامی در افراد بالای 60 سال فارسی‌زبان بود. مواد و روش‌ها: در این مطالعه مشاهده‌ای، 90 فرد سالمند بالای 60 سال (45 زن و 45 مرد) به دو گروه سالمند جوان (60 تا 74 سال) و سال‌خورده (75 تا 90 سال) و سه گروه تحصیلی (>5 سال، 6 تا 12 سال و >12 سال) تقسیم شدند. آزمون روانی کلامی در دو بخش روانی معنایی شامل نام‌بردن مقوله‌های حیوان و میوه و روانی واجی شامل نامیدن کلماتی که با واج‌های /ف/، /آ/ و /س/ شروع می‌شود، در مدت زمان 60 ثانیه اجرا شد. پاسخ افراد با ضبط‌صوت ثبت و نگارش شد و سپس با استفاده از روش ترویر مورد تجزیه‌وتحلیل قرار گرفت. داده‌های به‌دست‌آمده با استفاده آزمون غیرپارامتریک تی مستقل، تحلیل واریانس یک‌طرفه و ضریب همبستگی پیرسون تحلیل شد. یافته‌ها: یافته‌های حاصل از پژوهش نشان داد که تفاوت میان عملکرد روانی واجی و معنایی در بین دو جنس مذکر و مؤنث معنادار نیست، اما میانگین عملکرد روانی معنایی و روانی واجی در بین دو گروه سنی معنادار بود (P<0/001). همچنین بین سن و عملکرد انتقال همبستگی معکوس و معناداری به‌دست آمد (r=0/46 و P<0/01) و تحصیلات همبستگی مثبت و معناداری بر عملکرد انتقال نشان داد (r=0/36 و P<0/01). نتیجه‌گیری: نتایج حاصل از پژوهش نشان داد که عملکرد روانی معنایی و روانی واجی با افزایش سن کاهش و با افزایش تحصیلات افزایش می‌یابد، اما جنسیت بر آن تأثیری ندارد. به‌نظر می‌رسد لازم است در توانبخشی آسیب‌های زبانی در سالمندان نظیر زبان‌پریشی، موضوع افت توانمندی روانی کلامی در اثر افزایش سن درنظرگرفته شود.

Title: بررسی اثربخشی آموزش گروهی امیدمحور بر ارتقای کیفیت زندگی سالمندان

http://salmandj.uswr.ac.ir/article-1-1052-fa.html

اهداف: رشد سریع جمعیت به‌ویژه در کشورهای درحال‌توسعه موجب بروز پدیده سالمندی شده است؛ به‌گونه‌ای که در سال‌های کنونی پیرشدن جمعیت به یکی از مهم‌ترین مسائل بهداشت عمومی تبدیل شده است. این موضوع توجه به این گروه سنی را  اجتناب‌ناپذیر کرده است. اغلب مشکلاتی که سالمندان با آن رو‌به‌رو هستند، مشکلات جسمی و ناتوانی‌های حرکتی و مشکلات روحی-روانی است. ازجمله مسائل روحی-روانی که سالمندان با آن مواجه می‌شوند، ناامیدی، افسردگی و احساس تنهایی است. روانشناسان گام‌های بسیاری به‌منظور بهبود این علایم برداشته‌اند؛ ولی پس از ظهور جنبش روانشناسی مثبت، نظریه‌پردازان و محققان به‌جای توجه صِرف به تجارب یا ادراک‌های منفی به بررسی سازه‌هایی چون خودکنترلی، معنویت، شادی، خوش‌بینی و امید پرداختند. ازاین‌رو هدف اصلی پژوهش حاضر بررسی اثربخشی آموزش گروهی امیدمحور بر ارتقای کیفیت زندگی سالمندان است. مواد و روش‌ها: مطالعه حاضر نیمه‌آزمایشی با طرح پیش‌آزمون-پس‌آزمون با گروه کنترل بود. جامعه آماری این پژوهش را تمامی سالمندان زیرپوشش مرکز روزانه سالمندان امید گلستان در شهرستان مینودشت دربرمی‌گرفت. در این پژوهش نمونه‌گیری به روش دردسترس انجام شد؛ بدین‌صورت که از بین 60 نفر از سالمندان مراجعه‌کننده و داوطلب برای شرکت در جلسات گروهی امیدمحور، 30 نفر که واجد شرایط بودند، انتخاب و به‌صورت تصادفی در دو گروه 15 نفره آزمایش و کنترل تقسیم شدند. به‌منظور گردآوری داده‌ها از پرسش‌نامه فرم کوتاه 26 سؤالی کیفیت زندگی سازمان جهانی بهداشت استفاده شد. پس از اجرای پیش‌آزمون، اعضای گروه آزمایش به‌مدت هشت جلسه 90 دقیقه‌ای و هفته‌ای یک‌روز آموزش گروهی امید محور را آموختند و گروه کنترل آموزشی دریافت نکردند. سپس پس‌‌آزمون برای هر دو گروه تکرار شد. به‌منظور تجزیه‌وتحلیل نتایج، از روش تحلیل کواریانس در نسخه 16 نرم‌افزار SPSS استفاده شد. یافته‌ها: نتایج نشان داد که آموزش گروهی امیدمحور کیفیت زندگی در سالمندان گروه آزمایش را به‌طورمعناداری افزایش داده بود (0/05>P). همچنین نتایج نشان داد آموزش بُعد جسمانی، کیفیت زندگی سالمندان در گروه آزمایش را در مقایسه با گروه کنترل بهبود بخشیده بود (0/05>P). بُعد روانشناختی کیفیت زندگی گروه آزمایش نیز در قیاس با گروه کنترل تفاوت معناداری داشت (0/05>P). در ابعاد محیطی و اجتماعی نیز آموزش گروهی امیدمحور توانسته است کیفیت زندگی سالمندان گروه آزمایش را در مقایسه با گروه کنترل ارتقا دهد (0/05>P). نتیجه‌گیری: نتایج پژوهش حاضر نشان داد آموزش گروهی امید محور، افزایش کیفیت زندگی و ابعاد آن شامل ابعاد جسمانی، روانشناختی، محیطی و اجتماعی سالمندان را در پی دارد. ازاین‌رو با توجه به افزایش جمعیت سالمندان در جوامع درحال‌توسعه ازجمله ایران و لزوم توجه به سلامت جسمانی و روانشناختی این قشر می‌توان از روش آموزش گروهی امیدمحور  برای بهبود کیفیت زندگی سالمندان بهره برد.

Title: ادراک و باورها نسبت به سالخوردگی و تأثیر آن بر سلامت عمومی سالمندان: ارزیابی الگوی خودگردانی

http://salmandj.uswr.ac.ir/article-1-1003-fa.html

اهداف: باورها و ادراک‌ نسبت به سالخوردگی، تأثیر بسزایی بر بهزیستی و سلامت جمعیت بزرگسال دارد. یکی از مدل‌های نظری سودمند برای توضیح نقش ادراکات و باورها نسبت به سالخوردگی در تعیین برآیندهای سلامتی سالمندان، مدل خودگردانی است. مطالعه حاضر با هدف تعیین رابطه میان ادراک و باورها درباره سالمندی و سلامت عمومی سالمندان در قالب مدل خودگردانی انجام شد. مواد و روش‌ها: این مطالعه در قالب یک طرح توصیفی و با روش پیمایشی انجام شد. داده‌های مطالعه از 300 نفر از سالمندان شهر رشت (144 مرد و 156 زن) جمع‌آوری شد. نمونه‌ها با روش نمونه‌گیری دردسترس و از دو مکان آسایشگاه‌های سالمندان و خانواده‌ها انتخاب شده بودند. ابزار اندازه‌گیری عبارت بود از: پرسش‌نامه ادراک سالخوردگی (APQ) و پرسش‌نامه 28 سؤالی سلامت عمومی گلدبرگ. یافته‌ها: در این مطالعه 300 سالمند موردبررسی قرار گرفتند. همبستگی مثبت معناداری میان اکثر مؤلفه‌های ادراک سالخوردگی، یعنی خط زمانی مزمن و دوره‌ای، برآیندهای مثبت و منفی سالمندی، تظاهرات هیجانی و کنترل مثبت و منفی سالمندی با مؤلفه‌های سلامت عمومی سالمندان وجود داشت. تحلیل رگرسیون چندگانه سلسله‌مراتبی نشان داد که مؤلفه‌های ادراک سالمندی یعنی کنترل مثبت، کنترل منفی، برآیندهای منفی و خط زمانی دوره‌ای 31/2درصد از واریانس سلامت عمومی سالمندان را تبیین کرده است. نتیجه‌گیری: ادراک و باورها نسبت به سالخوردگی از تعیین‌کننده‌ها و پیش‌بین‌های اساسی سلامت عمومی در میان سالمندان است. مدل خودگردانی، چهارچوبی مناسب برای توضیح نقش باورها، ادراک‌ و فهم تجربیات سالخوردگی در زمینه سلامت جسمانی و روانی سالمندان است. توجه به نقش بالقوه ادراک سالمندی و نیز انجام مداخله‌های شناختی و رفتاری برای تعدیل نگرش‌ها دربرابر سالمندی با هدف ارتقای سلامت فیزیکی و روانی سالمندان ضروری به‌نظر می‌رسد.

Title: تأثیر خستگی عضلات دورکننده ران بر تعادل ایستا و پویای زنان سالمند

http://salmandj.uswr.ac.ir/article-1-927-fa.html

اهداف: کنترل پاسچر عبارت است از حفظ اندام‌ها و قسمت‌های مختلف بدن در راستای بیومکانیکی مناسب که به دو شکل ایستا و پویا وجود دارد. خستگی یکی از عواملی است که بر کنترل پاسچر و ثبات بدن تأثیر می‌گذارد. هدف از تحقیق حاضر، بررسی اثر خستگی عضلات دورکننده ران بر تعادل ایستا و پویای زنان سالمند است. مواد و روش‌ها: 25 زن سالمند سالم، بدون‌سابقه افتادن با میانگین سنی 8/5±60/24 سال، وزن 9/54±60/58 کیلوگرم و قد 7/65±153/42 سانتی‌متر در این مطالعه شرکت کردند. آزمودنی‌ها در جلسه اول آزمون‌های تعادل ایستا و پویا را در شرایط عادی (بدون خستگی) انجام دادند. در جلسه دوم ابتدا آزمودنی‌ها در برنامه خستگی عضلات دورکننده ران شرکت کردند و بلافاصله آزمون‌‌های تعادل ایستا و پویا را انجام دادند. به‌منظور مقایسه نتایج تحقیقات قبل و بعد از خستگی از آزمون تی وابسته استفاده شد. یافته‌ها: نتایج نشان داد برنامه خستگی عضلات دورکننده ران به‌طورمعنی‌‌داری نمرات آزمون‌‌های تعادل شامل تعادل ایستا و تعادل پویا را کاهش داده است. همچنین بین نمرات تعادل پویا و ایستا قبل و بعد از خستگی تفاوت معناداری وجود داشت و اندازه اثر برنامه خستگی در هر دو آزمون اندازه اثری بزرگ بود (0/05>P).  نتیجه‌گیری: کاهش نمرات آزمون‌‌های تعادل ایستا و پویا احتمالاً نشان‌دهنده‌ این است که خستگی عضلات دورکننده ران میزان آسیب و دفعات افتادگی در سالمندان را به‌موجب نداشتن تعادل افزایش می‌دهد.

Title: مقایسه تأثیر تعداد جلسات تمرینات آبی بر عوامل زیستی-حرکتی و تعادل سالمندان کم‌تحرک

http://salmandj.uswr.ac.ir/article-1-915-fa.html

اهداف: کاهش ظرفیت‌های جسمانی و عملکردی ازجمله عوامل خطرآفرین در افتادن، معلولیت، آسیب‌دیدگی یا مرگ سالمندان به‌شمار می‌رود. تمرین در آب یکی از روش‌های مطرح‌شده به‌منظور بهبود عملکرد سالمندان است. برای سالمندان انجام برخی از فعالیت‌ها در خشکی و مشکلات و خطرات آسیب‌دیدگی را به‌همراه دارد. با درنظرگرفتن دوره زمانی طولانی‌تر بازیافت در سالمندان و نبودِ امکان برخی سالمندان برای شرکت در فعالیت‌های بدنی به‌ویژه تمرین در آب، دست‌کاری تعداد جلسات تمرین در آب از اهمیت فراوانی برخوردار است. هدف این تحقیق مقایسه تأثیر تعداد جلسات تمرینات آبی در هفته بر شاخص‌های تعادلی و عملکردی سالمندان کم‌تحرک بود.  مواد و روش‌ها: پژوهش حاضر کاربردی و روش آن نیمه‌تجربی بود. در این مطالعه 36 مرد سالمند با میانگین سنی 2/15±64/36 سال، قد 4/32±165/05 سانتی‌متر، وزن 3/73±62/89 کیلوگرم و شاخص توده بدنی 1/72±23/12 مترمربع داوطلبانه انتخاب شدند. ارزیابی‌های اولیه پس از امضای فرم رضایت‌نامه شخصی انجام شد. سپس آزمودنی‌ها به‌صورت تصادفی در سه گروه 12نفری گروه دوجلسه‌ای و سه‌جلسه‌ای و گروه کنترل تقسیم شدند و برنامه تمرین آبی شش‌هفته‌ای اجرا شد. در آغاز و پایان برنامه تمرینات شاخص‌های تعادلی و عملکردی، قدرت اندام تحتانی و انعطاف‌پذیری موردارزیابی قرار گرفتند. برای بررسی اختلاف در پیش‌آزمون و پس‌آزمون هر گروه از آزمون تی زوجی و برای مقایسه نتایج در سه گروه با یکدیگر از آزمون آماری تحلیل واریانس یک‌طرفه (ANOVA) استفاده شد. همچنین تمامی عملیات آماری با استفاده از نسخه 17 نرم‌افزار SPSS در سطح معنی‌داری 0/05≥P محاسبه شد.  یافته‌ها: نتایج حاکی از آن است که در عامل‌های تعادل پویا و ایستا و توانایی حرکتی بین گروه دوجلسه‌ای و سه‌جلسه‌ای تمرین در هفته تفاوت معنی‌دار وجود ندارد (0/05≤P). ازسوی‌دیگر قدرت و انعطاف‌پذیری گروه سه‌جلسه‌ای در مقایسه با گروه دو‌جلسه‌ای تفاوت معنی‌داری نشان داد (0/05≥P). علاوه‌براین یافته‌های آزمون تی زوجی نشان داد در تمامی عامل‌ها بین میانگین نمرات پیش‌آزمون و پس‌آزمون هر دو گروه تجربی تفاوت معناداری وجود دارد (0/05≥P). بااین‌حال در گروه کنترل این تفاوت معنادار نبود (0/05≤P). درهرصورت در پس‌آزمون در مقایسه با پیش‌آزمون عوامل قدرت و انعطاف‌پذیری گروه سه جلسه تمرین آبی در هفته در مقایسه با گروه دو جلسه تمرین در هفته، افزایش به‌مراتب بیشتری را نشان داد. نتیجه‌گیری: به‌نظر می‌رسد در سالمندان کم‌تحرک  بعد از اجرای فعالیت‌های بدنی در  آب، بهبود عوامل جسمانی چون تعادل ایستا و پویا و توانایی حرکتی از حجم تمرین بیشتر از تعداد جلسات تمرینی تأثیر می‌پذیرد. البته افزایش تعداد جلسات تمرینی می‌تواند دیگر عوامل جسمانی از قبیل قدرت و انعطاف‌پذیری را به‌میزان مشابه یا بیشتری افزایش دهد.

Title: وضعیت رعایت اصول طراحی فراگیر در آسایشگاه‌های سالمندان شهر تهران

http://salmandj.uswr.ac.ir/article-1-1084-fa.html

اهداف: در سالیان اخیر روند سپردن سالمندان به آسایشگاه‌ها سیر صعودی داشته و آم‍اده‌س‍ازی‌ ف‍ض‍ایی‌‌ ک‍ه‌ پ‍اس‍خ‌گوی‌ ن‍ی‍ازه‍ای‌ ف‍ی‍زی‍ک‍ی‌، روان‌ش‍ن‍اخ‍ت‍ی‌، اج‍ت‍ماع‍ی‌ و م‍ع‍ن‍وی آنان باشد، ض‍رورت‌ پیدا کرده است. طراحی فراگیر به تولید محصولات یا ایجاد محیطی اطلاق می‌شود که در آن حداکثر امکان دسترسی برای همه افراد، اعم از سالمند و معلول، فراهم باشد. هدف این پژوهش تعیین وضعیت رعایت طراحی فراگیر آسایشگاه‌های سالمندان شهر تهران بود. مواد و روش‌ها: مطالعه حاضر از نوع توصیفی و جامعه پژوهش شامل تمامی آسایشگاه‌های سالمندان زیرپوشش بهزیستی شهر تهران در سال 1394 بود و برای انتخاب نمونه‌ها از روش نمونه‌گیری تمام‌شماری استفاده شد. اطلاعات با استفاده از چک‌لیست ارزیابی تناسب فیزیکی آسایشگاه سالمندان جمع‌آوری شد که شامل 12 خُرده‌مقیاس و 70 سؤال بود. روایی صوری و محتوایی این چک‌لیست قبلاً در مطالعه دیگری بررسی و تأیید شده بود. به‌منظور تجزیه‌وتحلیل اطلاعات از آمار توصیفی و بررسی شاخص‌های مرکزی استفاده شد. یافته‌ها: در بیشتر آسایشگاه‌ها وضعیت طراحی فراگیر به‌تفکیک تأمین ویژگی‌های کلی از نظر آشپزخانه (7/85درصد)، اتاق مدیریت (7/85درصد)، رختشوی‌خانه (9/92درصد)، وسایل گرمایشی و سرمایی (4/96درصد) از وضعیت مطلوبی برخوردار بود. این در حالی بود که تنها حدود یک‌سوم از آسایشگاه‌ها وسایل اطفای حریق داشتند. همچنین از نظر وجود نمازخانه و کتابخانه دردسترس و نیز از نظر وضعیت اتاق استراحت کارکنان بیشتر آنها وضعیت مطلوبی نداشتند. همچنین در اکثر آسایشگاه‌ها وضعیت طراحی فراگیر به‌تفکیک رعایت شاخص‌های تعریف‌شده در زیرمجموعه‌های آسایشگاه، از نظر طراحی فراگیر ورودی اصلی، اتاق درمان و توانبخشی و تا حدودی سرویس بهداشتی وضعیت مناسبی داشتند. بااین‌حال در بیشتر آنها اتاق نشیمن و سالن غذاخوری از نظر طراحی فراگیر وضعیت مطلوبی نداشتند. نتیجه‌گیری: یافته‌های این پژوهش نشان داد که اکثر آسایشگاه‌های سالمندان شهر تهران، براساس پرسش‌نامه تناسب فیزیکی آسایشگاه سالمندان، از اصول طراحی فراگیر تبعیت نمی‌کنند؛ بنابراین توصیه می‌شود ارگان‌های مسئول از طریق تدوین آیین‌نامه‌ها، برنامه‌ریزی‌ها و سیاست‌گذاری‌های دقیق‌تر در زمینه رعایت اصول طراحی فراگیر در محیط آسایشگاه‌ها، شرایطی فراهم کنند که امکان پاسخ‌دهی مناسب‌تر به نیازهای سالمندان فراهم شود.

Title: اثربخشی موسیقی‌درمانی هدفمند بر کیفیت خواب و غلبه بر بی‌خوابی در سالمندان

http://salmandj.uswr.ac.ir/article-1-992-fa.html

اهداف: هدف پژوهش حاضر تعیین اثربخشی موسیقی‌درمانی بر کیفیت کلی خواب و زیرمقیاس‌های آن در سالمندان مبتلا به اختلال بی‌خوابی اولیه بوده است. مواد و روش‌ها: پژوهش حاضر از نوع آزمایشی بود و جامعه آماری آن را 5000 سالمند عضو کانون جهان‌دیدگان شیراز تشکیل می‌دادند. نمونه‌های این مطالعه 44 نفر از سالمندانی بودند که به‌طورتصادفی انتخاب شدند و با احتساب شرایط ورود به مطالعه و خروج از آن در دو گروه آزمایش موسیقی‌درمانی و کنترل قرار گرفتند. به‌دنبال ریزش 6/82درصدی، 41 نفر سالمند با میانگین سنی 68/88 سال با شاخص کیفیت خواب پیتزبورگ در پیش‌آزمون، پس‌آزمون و پیگیری ارزیابی شدند که به بی‌خوابی اولیه مبتلا بودند. در این مطالعه، مداخله چهارهفته‌ای (دوبار در هفته) با روش موسیقی‌درمانی هدفمند شامل درون‌مایه‌های موسیقی‌ آرام‌بخش، فرح‌بخش و نیروبخش برای کاهش اضطراب شبانه و سرحالی و سرزندگی روزانه انجام شده است. برای تجزیه‌وتحلیل داده‌ها نسخه 21 نرم‌افزار SPSS و روش‌های آماری تحلیل کوواریانس تک و چندمتغیره به‌کاررفت. یافته‌ها: میانگین نمرات کیفیت کلی خواب قبل از مداخله در گروه‌های آزمایش و کنترل به‌ترتیب 13/31 و 12/95 بود که پس از مداخله در پس‌آزمون به 7/59 و 9/73 و در پیگیری سه ماه بعد 7/22 و 9/32 تغییر یافته است. تفاوت میانگین نمرات کیفیت کلی خواب پس از مداخله، از نظر آماری معنادار بوده است (0/001>P). نتیجه‌گیری: مطالعه حاضر نشان داد که موسیقی‌درمانی هدفمند می‌تواند کیفیت کلی خواب، کیفیت ذهنی خواب، عملکرد روزانه و کاهش مصرف داروهای خواب‌آور را در سالمندان مبتلا به اختلال بی‌خوابی بهبود بخشد. متخصصان موسیقی‌درمانی و بهداشت روان در حیطه سالمندی توصیه می‌شود که از این روش استفاده کنند.

Title: پایگاه‌های اطلاعاتی و مجلات سالمندی: بررسی مقایسه‌ای پایگاه‌های اطلاعاتی ازنظر پوشش مجلات سالمندی

http://salmandj.uswr.ac.ir/article-1-1058-fa.html

اهداف: پایگاه‌های اطلاعاتی و مجلات زیرپوشش آن‌ها، جزو مهم‌ترین منابع اطلاعاتی دانشجویان و متخصصان حوزه‌ها و رشته‌های گوناگون از جمله سالمندی است. با توجه به تعدّد پایگاه‌های اطلاعاتی و هزینه اشتراک بالای آن‌ها، آگاهی از پایگاه‌هایی که بیشترین مجلات مربوط به حوزه سالمندی را پوشش می‌دهد، برای کتابخانه‌ها و متخصصان و دانشجویان این رشته ضروری است.  مواد و روش‌ها: روش پژوهش حاضر تحلیل محتوای کمّی و نوع پژوهش کاربردی بود. جامعه این پژوهش را 177 مجله الکترونیکی علمی فعال تشکیل می‌داد که با جستجوی کلیدواژه موضوعی سالمندی از پایگاه اولریخ استخراج شده بود. به‌منظور تجزیه‌و‌تحلیل داده‌ها نرم‌افزار اکسل و نسخه 19 نرم‌افزار SPSS به‌کاررفت و آمار توصیفی و درصد همپوشانی سنتی 14 پایگاه و سرویس نمایه‌سازی نیز بررسی شد. یافته‌ها: نتایج پژوهش نشان داد که پایگاه پابمد با پوشش 109 مجله (61/58درصد) بیشترین پوشش مجلات حوزه سالمندی را دارد. در میان ناشران مجلات، الزویر با انتشار 15 مجله (8/5درصد) در رتبه اول قرار گرفت. علاوه‌براین بزرگ‌ترین تأمین‌کننده متن کامل مقالات اِبسکوهاست با پوشش 104 مجله (58/75درصد) بوده است. بیشترین همپوشانی از نظر مجلات زیرپوشش، میان دو پایگاه کارِنت کانتنس و وب آف ساینس با درصد همپوشانی 82/09 بوده است. نتیجه‌گیری: یافته‌های پژوهش نشان داد که کاربران برای جست‌وجو و تأمین‌کنندگان اطلاعات برای کاربران (مانند کتابخانه‌ها) برای اشتراک پایگاه‌های اطلاعاتی و کاهش هزینه‌ها می‌توانند پایگاه‌هایی را جایگزین یکدیگر کنند که همپوشانی بالا با یکدیگر دارد.

Title: پاسخ‌های اندوتلین-1 و فشارخون زنان یائسه به تمرینات هوازی

http://salmandj.uswr.ac.ir/article-1-1047-fa.html

اهداف: اندوتلین-1 تنگ‌کننده قوی عروق است که به‌وسیله سلول‌های اندوتلیال عروقی ترشح و به‌عنوان قوی‌ترین تنگ‌کننده عروقی شناخته می‌شود.  هدف این تحقیق مطالعه تأثیر یک دوره تمرین هوازی بر غلظت اندوتلین-1 پلاسما و رابطه آن با فشارخون زنان یائسه بود. مواد و روش‌ها: 20 زن یائسه با میانگین سن 5/67±67/85 سال، قد 7/7±153/50 سانتی‌متر، وزن 11/96±66/16 کیلوگرم، شاخص توده بدنی 4/98±28/15 مترمربع، درصد چربی 3/65±18/41 و نسبت دور کمر به لگن 0/04±0/92 سانتی‌متر به‌صورت هدفمند انتخاب و به‌طورتصادفی به دو گروه 10 نفره کنترل و تجربی تقسیم شدند. گروه تجربی هشت هفته (سه روز در هفته) تمرین هوازی با شدت 60 تا 70 درصد حداکثر ضربان قلب را انجام داد. قبل و بعد از هشت هفته تمرین، سطح استراحتی غلظت اندوتلین1- فشارخون سیستولیک و دیاستولیک اندازه‌گیری و ثبت شد. برای بررسی تغییرات درون‌گروهی از آزمون تی همبسته و تفاوت‌های بین‌گروهی از آزمون تی مستقل و برای بررسی رابطه بین اندوتلین و فشارخون از ضریب همبستگی پیرسون استفاده شد. سطح معنی‌داری 0/05>t درنظرگرفته شد.  یافته‌ها: نتایج این تحقیق نشان داد یک دوره تمرین هوازی تأثیر معنی‌داری بر کاهش غلظت اندوتلین-1 پلاسمای زنان یائسه دارد (0/005=P). همچنین یک دوره تمرین هوازی بر کاهش فشارخون سیستولیک (0/003=P) و دیاستولیک (0/002=P) زنان یائسه تأثیر معنی‌داری می‌گذارد و بین غلظت اندوتلین-1 و فشارخون سیستولیک همبستگی مستقیمی وجود دارد (0/59=P). بااین‌حال بین غلظت اندوتلین-1 و فشارخون دیاستولیک همبستگی مشاهده نشد (0/39=r).  نتیجه‌گیری: با توجه به کاهش غلظت اندوتلین-1 پلاسما و کاهش فشارخون سیستولیک و دیاستولیک بر اثر تمرین هوازی و با توجه به رابطه اندوتلین-1 با فشارخون سیستولیک به‌نظر می‌رسد یک دوره تمرین هوازی با این شدت و حجم می‌تواند در کاهش فشارخون سیستولیک و دیاستولیک و نیز کاهش غلظت اندوتلین-1 پلاسما به‌عنوان عامل خطر ایجاد پرفشاری خون زنان یائسه مؤثر باشد.
